# Hypertension and Alcohol: A Mechanistic Approach

**DOI:** 10.7759/cureus.10086

**Published:** 2020-08-27

**Authors:** Onosetale M Okojie, Faheem Javed, Lawman Chiwome, Pousette Hamid

**Affiliations:** 1 Family Medicine, California Institute of Behavioral Neurosciences & Psychology, Fairfield, USA; 2 Anaesthesia, California Institute of Behavioral Neurosciences & Psychology, Fairfield, USA; 3 General Internal Medicine, California Institute of Behavioral Neurosciences & Psychology, Fairfield, USA; 4 Neurology, California Institute of Behavioral Neurosciences & Psychology, Fairfield, USA

**Keywords:** hypertension, alcohol, secondary hypertension, insulin resistance

## Abstract

Hypertension is a global public health challenge and a major cause of morbidity and mortality. Alcohol is one of the most frequently abused substances around the world. The financial implications of treating hypertension are also significant. Developing successful strategies to prevent hypertension may be as important, if not more important, than managing the disease once it arises. In this review we assess the relationship between alcohol use and hypertension development. We have searched the available literature using the PubMed database and identified studies that discussed the relationship between alcohol use and either primary or any of the common causes of secondary hypertension. We found that heavy alcohol use was almost invariably associated with increased risk of developing primary hypertension, regardless of the age or gender of the participants. The relationship between low or moderate alcohol use and hypertension is less clear and some evidence even points towards possible protective effects. The pathophysiology behind the association is incompletely understood and a number of mechanisms have been proposed. Heavy alcohol use also increases the risk of insulin resistance and obstructive sleep apnea, known causes of secondary hypertension. It has also been linked to a state of hypercortisolism, sometimes called pseudo-Cushing state. Moderate alcohol consumption may be protective against diabetes and hyperthyroidism associated with Graves' disease. Overall, public health efforts should address the issue of heavy alcohol use. There does not appear to be enough evidence to recommend abstinence to those consuming low amounts of alcohol with the aim of protecting against hypertension. We believe that the current understanding of the issue is insufficient and that more both basic science and clinical research needs to be done.

## Introduction and background

Hypertension is one of the most common conditions diagnosed globally and one of the most important causes of early mortality. Almost half of US adults have been diagnosed with hypertension and only about a quarter of them have it under control. Considering that hypertension is generally asymptomatic, it is likely that the real number is higher as many may not have been diagnosed. Among those with poorly controlled hypertension, almost half either have not been prescribed any treatment or do not take it. The Center for Disease Control (CDC) reports that hypertension is at least partly responsible for up to 1,300 deaths per day in the United States [1]. It is well known that hypertension is a major risk factor for cardiovascular, cerebrovascular, and renal disease, all of which are among the 10 most frequent causes of death in the US [2]. Hypertension is, therefore, a major public health topic and developing strategies for combating hypertension has been the focus of a number of public health campaigns. The costs associated with hypertension are substantial, both in terms of management of hypertension alone (life-long medications) and in terms of the management of complications [3]. The number of days patients are absent from work due to conditions associated with hypertension is also significant. Currently, the US spends an average of $131 billion each year on hypertension [4]. This figure is likely to increase in the future and does not include all the costs associated with hypertension management. Such high costs associated with management as well as the risk of non-compliance make acting on modifiable risk factors an essential part of any public health strategy focused on hypertension.

Alcohol is one of the most commonly abused substances worldwide. In the United States, around a quarter of the population reported engaging in binge drinking over the past month and almost 6% of adults can be diagnosed with alcohol use disorder (defined as a chronic brain disease characterized by inability to control alcohol use despite social or health consequences). Alcohol abuse is not only a major cause of liver disease, but also an important risk factor for cancers of the esophagus, larynx, mouth, liver, and breast [5]. It is frequently reported that moderate alcohol consumption is associated with higher high-density lipoprotein (HDL) levels and that it may decrease the risk of coronary artery disease (CAD) development [6]. Nevertheless, the evidence of cardioprotection is weak and due to its significant associations with other conditions, alcohol should not be used for this indication [7].

Currently, there is little data on the association between alcohol use and hypertension. Considering the high prevalence of both alcohol use and hypertension, as well as hypertension’s possible association with other cardiovascular risk factors, a link between the two is likely. Finding that link could prove to be vitally important as public health measures aimed at alcohol abuse could impact hypertension rates, and subsequently cardiovascular morbidity and mortality.

For this article, we have searched the currently available literature using the PubMed database. No quality assessment of individual papers was performed.

## Review

Primary hypertension

Taking into account the high incidence of alcohol abuse and hypertension, and the potential associations between alcohol consumption and other risk factors for cardiovascular disease, it is safe to conclude that there is a pathophysiological link between the two conditions. Hypertension is clearly associated with lifestyle, and various lifestyle modifications have been found to contribute to the reduction in blood pressure. Some of them are weight loss, physical activity, and changes in diet. The pathophysiology of atherosclerosis and primary hypertension is complex and incompletely understood. Therefore, identifying risk factors if essential in developing management strategies.

Hering et al. found that the baseline muscle sympathetic nerve activity (MSNA) was significantly elevated in hypertensive patients compared to healthy controls [8]. Alcohol was found to raise resting heart rate and MSNA compared to placebo [9]. In normotensive individuals, alcohol was not associated with blood pressure changes, although MSNA increased. The opposite was seen in hypertensive patients - alcohol was associated with significant increases in blood pressure but no effect on MSNA was observed [8]. Huntgeburth et al. also found alcohol to be significantly associated with blood pressure increases [10]. Beijin reported that the effect of alcohol abuse is additive to that of obesity. He also found that the effects were partly reversible [11]. It is possible that the effect may differ between men and women. Lui et al. reported that a history of weekly heavy drinking elevated the risk of hypertension in women but not in men. It should be noted that the population studied by Lui et al. was younger (up to the age of 55) and may therefore not be generalizable to the whole population [12]. Ji et al. found alcohol dependence to be significantly associated with both systolic and diastolic blood pressure elevations (p<0.01), with the odds ratio (OR) being higher the greater the alcohol consumption among Chinese patients with newly diagnosed hypertension. The study only included Chinese patients with newly diagnosed hypertension [13]. Briasoulis et al. found no statistically significant increase in the risk of hypertension development among men who were classified as light or moderate drinkers. They did, however, find a significant increase in risk with heavy alcohol consumption as the relative risk (RR) was 1.61 (CI 1.38-1.87, p<0.001). Among women, light drinking was associated with significantly lower risk of hypertension development (RR 0.87; 95% CI 0.82-0.92; p<0.001), no significant association was found with moderate consumption, and a significantly increased risk with heavy consumption (RR 1.19; 95% CI 1.07-1.32; p<0.002). Heavy consumption, which led to significant increases in the risk of hypertension development, was defined as consumption of at least 31 g/d irrespective of the baseline characteristics [14]. Puddley et al. said that the claim that light alcohol intake was protective in women can no longer be upheld [15]. Kawano reported that drinking later in the day was associated with elevations in daytime and reductions in nighttime blood pressure, leading to no significant change in 24-hour blood pressure [16].

The pathophysiology of the relationship between alcohol intake and blood pressure is likely multifactorial and multiple different mechanisms have been proposed. The most intuitive one relates to the effect of alcohol on cholesterol, and consequently on atherosclerosis development and some authors believe that this explains the possible blood pressure reductions among light drinkers [16]. The possible mechanisms behind the association with blood pressure increases include endothelial dysfunction, intracellular calcium accumulation, stimulation of the renin-angiotensin-aldosterone system, elevated sympathetic activity, vasoconstriction, and elevated oxidative stress [15,17-19]. Wakabayashi et al. believe that ethanol directly acts on blood pressure by mobilizing both intra- and extracellular calcium, and therefore, simultaneously has two opposing effects. Alcohol also appears to impair endothelial vasodilation mediated by nitric oxide and endothelium-derived hyperpolarizing factor [18]. As such, Husain et al. believe that besides physical exercise, angiotensin-converting enzyme (ACE) inhibitors/angiotensin receptor (AR) blockers and calcium-channel inhibitors are best suited for managing hypertension associated with excessive alcohol intake [19].

Overall, all the studies we found point towards elevated risk of primary hypertension development among heavy drinkers regardless of gender. The risk among those who consume low and moderate amounts of alcohol may not be significantly elevated. Some studies even suggest that non-heavy alcohol consumption may be protective. It should be noted that not all the aforementioned findings can be generalized to the total population as the patient sample that was used was limited, either geographically or age-wise. Moreover, the pathophysiology of the phenomenon remains incompletely understood. In order to further our understanding of the issue, it would be essential to do more research on the pathophysiology of alcohol-induced hypertension. Also, a larger cohort study with a more generalizable patient population and properly identified confounders is needed to assess the relationship between low and moderate alcohol intake and hypertension development.

Secondary hypertension

Excessive alcohol consumption is known to be associated with a number of other medical conditions, which may in turn lead to secondary hypertension development. Alcohol withdrawal also tends to trigger a severe sympathetic response, consequently leading to dangerously high blood pressure levels. Furthermore, long-term alcohol consumption has been linked to insulin resistance, which is a known risk factor for hypertension development [18]. Alcohol is highly caloric and excessive alcohol consumption can precipitate obesity and lead to obesity-related complications, including hypertension. Although the evidence of direct nephrotoxicity is lacking, many mechanisms of kidney injury associated with alcohol consumption have been proposed, including oxidative stress and renal disease caused by other systemic conditions [20].

Leal et al. found that heavy alcohol consumption led to renal atrophy in rats. The finding was not reversible even after long-term withdrawal [21]. Any cause of renal disease may lead to hypertension. Alcohol intake has also been associated with hypercortisolism, sometimes referred to as pseudo-Cushing state [22]. Elevated levels of cortisol lead to secondary hypertension. According to Taveira et al., alcohol use significantly increases the odds ratio of developing obstructive sleep apnea, a known cause of secondary hypertension (OR 1.33, 95% CI 1.10-1.62) [23]. On the other hand, Knott et al. did a systematic review of 38 observational studies, involving 1.9 million participants and found that moderate levels of alcohol consumption may lead to decreases in risk of diabetes type 2 development [24]. Carle et al. also reported that moderate alcohol consumption was associated with significantly decreased risk of developing Graves' disease with hyperthyroidism [25].

The effect of alcohol on the development of secondary hypertension is also difficult to assess. Renal disease, diabetes, obstructive sleep apnea, hypercortisolism, and hyperthyroidism are among the most common causes of secondary hypertension. Alcohol use was found to be significantly associated with obstructive sleep apnea, possibly as a result of the high-calorie nature of alcoholic drinks, which may lead to obesity. Alcohol use may also be associated with hypercortisolism, another possible cause of secondary hypertension, while it is likely protective against hyperthyroidism-induced hypertension. The association between alcohol and renal disease and diabetes is less clear. Although heavy alcohol use leads to insulin deficiency, moderate alcohol use has been linked to decreases in diabetes risk. The evidence of kidney damage in humans in relation to alcohol use is limited and most of the proposed mechanisms are based on the current understanding of human pathophysiology and on animal models. In order to get a clearer picture of the possible associations between alcohol use and secondary hypertension, more research needs to be done.

Alcoholic drink equivalent refers to any beverage containing 0.6 oz or 14 grams of pure alcohol in the US [26-28]. It is important to understand this as it encourages drinking sensibly and in moderation as there is no drink of moderation. Table 1 shows the alcoholic drink equivalents in the US.

**Table 1:** Alcoholic drink equivalents.

Beer 5% alcohol	Table wine 12% alcohol	Malt liquor 12% alcohol	Hard liquor 40% alcohol
12 oz = 1 drink	5 oz = 1 drink	12 oz = 1.5 drinks	a shot (1.5 oz) = 1 drink
16 oz = 1.3 drinks	25 oz = 5 drinks	16 oz = 2 drinks	half pint (6.8 oz) = 4.5 drink
20 oz = 2 drinks		22 oz = 2.5 drinks	a pint (12.7) = 8.5 drinks
40 oz = 3.3 drinks		40 oz = 4.5 drinks	A fifth (24.5) = 17 drinks

A drink is defined as 14 g of pure alcohol - a reliable way to know how much alcohol has been consumed [29-30]. Table 2 and Table 3 show the drinking level of alcohol and the associated risk in males and females.

**Table 2:** Drinking level of alcohol and risk in males. USS = US Standard

Drinking level	Drinks per day (g)	Drinks per day (USS)	Drinks per week (USS)
low risk	1 to 40 g	0 to 3 drinks	0 to 20 drinks
medium risk	41 to 60 g	3 to < 4 drinks	21 to 30 drinks
high risk	61 to 100 g	4 to 7 drinks	31 to 50 drinks
very high risk	101+ g	7+ drinks	51+ drinks
binge	>70 g in one occasion	>5 drinks in 2 hr	-

**Table 3:** Drinking level of alcohol and risk in females. USS = US Standard

Drinking level	Drinks per day (g)	Drinks per day (USS)	Drinks in week (USS)
low risk	1 to 20 g	0 to 1 drinks	0 to <10 drinks
medium	21 to 40 g	2 to <3 drinks	10 to <20 drinks
high	41 to 60 g	3 to <4 drinks	20 to 30 drinks
very high	61+ g	4+ drinks	31+ drinks
binge	>56 g in one occasion	>4 drinks in 2 hr	

What is unique about this article

The data suggest that the prevalence of hypertension might reduce if the number of heavy drinkers was reduced [15]. At this moment it is impossible to tell if hypertension resolves following cessation of alcohol abuse. We also found no evidence on whether antihypertensives could be discontinued following successful alcohol withdrawal. If antihypertensives can be safely discontinued in some of those patients and not in others, it raises the question as to what the mechanism behind the phenomenon and whether it depends on more than just the baseline blood pressure reading. Moreover, no studies we have found have properly addressed the types of beverages the individuals consumed. If there is discrepancy, it needs to be determined if a substance other than ethanol may be responsible for the blood pressure changes. These are some of the questions that remain unanswered, and it is our hope that they are addressed in future studies.

Limitations

No quality assessment of individual studies used for this review was performed. Findings in some of the studies may not be applicable to the general population as the sample size was not representative of the entirety of the population. The evidence in many of the studies is weak and the conclusions were often based on animal models. Performing randomized controlled trials would be unethical as it would require intentionally exposing participants to a substance that is known to be harmful.

## Conclusions

In this review, we have tried to address the relationship between alcohol abuse, known to cause significant metabolic derangements, and hypertension, one of the most prevalent diseases worldwide. Heavy alcohol use is generally seen as a significant risk factor for essential hypertension development. The risk is less clear for those who consume low and moderate levels of alcohol. It is unclear if the effect is equivalent in males and females. The possible pathophysiologic mechanisms involve endothelial dysfunction, vasoconstriction, sympathetic activation, and activation of the renin-angiotensin-aldosterone system. Alcohol use is also associated with some known causes of secondary hypertension, such as obstructive sleep apnea, and, possibly, renal disease. Alcohol is also associated with diabetes although moderate drinking may be protective. More research is needed in the future, including systematic reviews addressing the most common causes of secondary hypertension.
